# Commentary: Effects of Age and Initial Risk Perception on Balloon Analog Risk Task: The Mediating Role of Processing Speed and Need for Cognitive Closure

**DOI:** 10.3389/fpsyg.2016.01320

**Published:** 2016-08-29

**Authors:** Lukasz Walasek

**Affiliations:** Psychology, University of WarwickCoventry, UK

**Keywords:** aging, risk-taking, balloon analog risk task (BART), cognitive modeling, dual-system theories

Existing research strongly suggests that age-related changes in the cognitive system influence preferential choice. While the reduction of fluid cognitive ability can lead to sub-optimal decision outcomes (Finucane et al., 2000), experience garnered during one's lifespan can also improve one's decision making (Mata et al., 2007; Bruine de Bruin et al., 2014). How can research on aging and decision making explain such mixed results? A reasonable approach is to adhere to a clear definition of optimality in choice behavior, which must be grounded in principles of cognitive psychology. Indeed, this approach has led many researchers to identify distinct cognitive processes that may be responsible for suboptimal decisions among older adults. Among many, these include memory (Buckner, 2004), perception (Schneider and Pichora-Fuller, 2000), and executive functions (Schiebener and Brand, 2015).

In this special issue, Koscielniak et al. (2016) focus on the effect of aging on risky choice. Comparing the performance of two age groups, they found that older female adults exhibit a lower propensity to take risks on the Balloon Analog Risk Task (BART). At the same time, their results showed that both younger and older females adapt to initial failures and successes and are capable of adjusting their risk taking behavior over the course of repeated trials. Koscielniak et al. (2016) positioned their findings within the broader framework of the dual-system reasoning, attributing the overall poor performance of older adults to a “decline in deliberative processes” (p. 6).

In this commentary, I offer a cautionary note about the reliance on such theoretical frameworks to make predictions about the cognitive underpinnings of preferential choice. While the conclusions drawn by the authors are supported by their data, the use of verbal (i.e., not quantitative, Lewandowsky and Farrell, 2011) theories can lead to erroneous inferences. Instead, future advances should rely on cognitive modeling to decouple competing mechanisms that can give rise to differences in choices between younger and older adults. In challenging verbal and descriptive (i.e., statistical) models in study of risky choice and aging, many now argue that cognitive modeling removes ambiguity associated with the interpretation of cognitive processes (Lewandowsky and Farrell, 2011). In the case of the BART itself, several models have been proposed, each specifying specific cognitive mechanisms behind decisions to either pump or to secure one's earnings (Wallsten et al., 2005; Schmitz et al., 2016). In a 4-parameter version of the model by Wallsten et al. (2005), for example, α and μ parameters control the learning rate at which one's belief that a balloon will burst on a given trial is updated, γ^+^ represents the general propensity to take risks, and β captures the behavioral consistency of the agents. Crucially, Wallsten and colleagues showed that parameters recovered in their study correlated with self-reported indices of risky behaviors, supporting the view that their specification of the model captures the cognitive components of risk taking in BART (but see a discussion of alternative models by van Ravenzwaaij et al., 2011). In fact, research has found that the differences between young and old on BART performance can be attributed to heightened reward-sensitivity and the initial perception of risk (Cavanagh et al., 2012), as opposed to differences in the ability to update beliefs based on observed outcomes (Rolison et al., 2012). These results are consistent with the efforts of Koscielniak et al. (2016), but the results of model fitting present a clear advantage over verbal theories for a number of reasons.

First, interpretable parameters of the model applied to BART can be used to draw parallels with other areas in which the effect of aging has been studied. Comparisons with performance on other risk tasks (and of other populations) can produce converging evidence about the cognitive processes involved. This is particularly important as it reduces the chance that a particular paradigm (e.g., BART) becomes the subject of empirical research in itself. Second, cognitive modeling can lead to alternative interpretations concerning risky choice. To illustrate this point, consider a widely held belief about the negative correlation between cognitive ability and risk aversion (e.g., Dohmen et al., 2010). Recent findings by Andersson et al. (2013) showed that this association is in fact spurious and simply reflects an increased rate of random choice among those with lower cognitive ability. Although the authors did not use cognitive modeling to illustrate their finding, it is easy to see that in many cases such mistakes could be avoided if a correctly specified model with a noise parameter was fit to the data. In the context of BART and aging, existing models can discern between noisiness of responding and risk preferences, and they are therefore well equipped to recover such patterns. Indeed, previous studies have found that older adults are less consistent in their decisions (Finucane et al., 2005). Finally, cognitive models can be extended to study dynamic aspects of cognition. Much work has applied drift diffusion models to understand cognitive performance of younger and older adults on a range of tasks (Ratcliff et al., 2006; McKoon and Ratcliff, 2013). Such models take into account response times and can therefore tell us more about the time-accuracy tradeoffs involved in a decision process. This modeling approach is particularly suitable for studying the effects of aging, as older participants often adapt their strategies to account for the decline in their fluid cognitive ability (Smith and Brewer, 1995).

In sum, Koscielniak et al. (2016) contribute to the understanding of how cognitive and motivational factors influence preferential choice at different ages. This commentary highlights the fact that future research can build on these findings using cognitive modeling techniques to identify specific aspects of the cognitive process that impact risk preferences among young and old adults. Such efforts correspond to a shift from verbal and descriptive models toward quantitative models of cognition.

## Author contributions

The author confirms being the sole contributor of this work and approved it for publication.

## Funding

This work was supported by Leverhulme Trust Grant RP2012-V-022.

### Conflict of interest statement

The author declares that the research was conducted in the absence of any commercial or financial relationships that could be construed as a potential conflict of interest.
